# Identification of a Broad-Spectrum Viral Inhibitor Targeting a Novel Allosteric Site in the RNA-Dependent RNA Polymerases of Dengue Virus and Norovirus

**DOI:** 10.3389/fmicb.2020.01440

**Published:** 2020-06-25

**Authors:** Dongrong Yi, Quanjie Li, Lili Pang, Yujia Wang, Yongxin Zhang, Zhaojun Duan, Chen Liang, Shan Cen

**Affiliations:** ^1^Department of Immunology, Institute of Medicinal Biotechnology, Chinese Academy of Medical Sciences, Beijing, China; ^2^National Institute for Viral Disease Control & Prevention, Chinese Center for Disease Control and Prevention, Beijing, China; ^3^Lady Davis Institute, Jewish General Hospital, McGill University, Montreal, QC, Canada; ^4^CAMS Key Laboratory of Antiviral Drug Research, Chinese Academy of Medical Sciences, Peking Union Medical College, Beijing, China

**Keywords:** antiviral agents, re-emerging viruses, dengue virus, norovirus, RNA-dependent RNA polymerase, virus replication, structure-based virtual screening, allosteric site

## Abstract

All RNA viruses encode the RNA-dependent RNA polymerase (RdRp) which replicates and transcribes viral RNA. This essential viral enzyme does not exist in mammalian cells, thus presents a main target for the development of antiviral drugs with potential pan-antiviral activity. In this study, we take advantage of the structurally equivalent site in the dengue virus (DENV) RdRp, the N-pocket, and in the human norovirus (hNV) RdRp, the B-site, and performed a parallel structure-based virtual screening to discover compounds that can inhibit the RdRps of both hNV and DENV. We successfully identified a small molecule called Entrectinib (RAI-13) as a potent inhibitor of both hNV and DENV infection. Specifically, RAI-13 binds directly to hNV and DENV RdRps, effectively inhibits the polymerase activity in the *in vitro* biochemical assays, and exhibits does-responsive inhibition of murine norovirus (MNV) and DENV2 infection with IC50 values of 2.01 and 2.43 μM, respectively. Most promisingly, RAI-13 inhibits hepatitis C virus (HCV) infection by 95% at the 2 μM concentration. We have therefore discovered a small molecule compound that targets an allosteric site that is shared by different viral RdRps and strongly inhibits multiple pathogenic RNA viruses, thus holding the potential of being developed into a broad-spectrum antiviral drug.

## Introduction

The frequent emergence of new viral pathogens has become a severe threat to global population health. The emerging viruses such as human noroviruses (hNV) and Dengue viruses (DENV) are highly contagious and spread worldwide each year (Zheng et al., 2006; Robilotti et al., 2015; Wilder-Smith et al., 2019). Norovirus infection leads to intense diarrhea, vomiting, and stomach pain, and causes up to 200,000 deaths every year (Patel et al., 2008; Glass et al., 2009). Dengue fever is a widespread mosquito-borne disease that causes fever, rash, joint pain, and severe hemorrhagic shock (Whitehorn and Simmons, 2011). An approximated 390 million dengue infections occur worldwide every year, with up to 96 million resulting in sickness (Bhatt et al., 2013). Effective antivirals and vaccines are the tools to control and prevent pathogenic viral infections. In addition to virus-specific drugs, broad-spectrum antiviral drugs are deemed valuable to quickly respond to new viral pandemics (Adalja and Inglesby, 2019).

RNA viruses all encode a crucial enzyme, RNA-dependent RNA polymerase (RdRp) (Ahlquist, 2002). Despite the sequence diversity of RdRp across virus families, these viral RdRps share a structurally homologous core that resembles a right-hand composed of the palm, fingers, and thumb domains (Bressanelli et al., 1999), which provides a rational basis for broad-spectrum drug design. There are two main types of binding pockets for inhibitors in RdRp: the catalytic active site and the allosteric binding sites (Brown et al., 2016). Nucleos(t)ide inhibitors (NIs) such as BCX4430 (Warren et al., 2014), favipiravir (Furuta et al., 2009), ribavirin (Crotty et al., 2000), and remdesivir (Brown et al., 2019) exhibit broad-spectrum antiviral activity by targeting the polymerase active site. After being phosphorylated to the triphosphate form, these antivirals are incorporated into the growing viral RNA strand and lead to chain termination. However, the inhibition mode of NIs often causes off-target side effects. In contrast, non-nucleoside inhibitors (NNIs) bind to the allosteric pockets and exhibit antiviral activity through preventing conformational changes that are required for viral RNA transcription (Sofia et al., 2012). Due to the low toxicities and low side effects, NNIs have gained great interest in the development of antiviral drugs. Until now, most of the NNIs that are in clinical use or undergoing clinical trials are developed against HCV and are virus-specific (Membreno and Lawitz, 2011; Gerber et al., 2013; Kayali and Schmidt, 2014; Andersen et al., 2020).

As the most extensively studied flavivirus, five allosteric sites have been identified for HCV NNIs, including thumb site I, thumb site II, palm site I, palm site II, and palm site β. Among them, the palm site I is reported to be structurally equivalent to the binding pose, called N-pocket, identified for allosteric DENV RdRp inhibitors (Lim et al., 2016; Noble et al., 2016). In HCV RdRp, the palm site I located in the inner thumb/palm domain and surrounded by the primer grip and β-hairpin loop on both sides. Palm I inhibitors, such as dasabuvir (ABT-333) (Kati et al., 2015), RG7790 (setrobuvir) (Kapelusznik et al., 2012), and ABT-072 (Randolph et al., 2018), bind to this region and block conformational changes that are needed for *de novo* initiation. Similarly, the N-pocket in DENV RdRp is also situated at the interface of the thumb and palm subdomains and is adjacent to the priming loop (aa782–809), which is equivalent to the β-hairpin loop (residues 443–455) in HCV RdRp. The crystal structures of DENV RdRp in complex with the N-pocket inhibitors, including compounds 27, compound 29, and JF-31-MG46, PC-79-SH52, and FD-83-KI26, revealed that the ligands form hydrogen bond with priming loop residue T794 and bind into the N-pocket that consist of residues L511, L514, C709, S710, H711, R729, R737, M761, M765, T766, and 793–803 (Lim et al., 2016; Noble et al., 2016). Among them, R729 and R737 are reported to interact with the substrates bound in the active site, and play a similar function as R394 and R386 in HCV RdRp (Lim et al., 2016).

Interestingly, a similar NNI binding pose has also been identified in hNV RdRp. Delia Tarantino et al. reported the 3D structure of hNV RdRp/PPNDS complex and pointed out that the binding site of PPNDS, called B-site, is structurally equivalent to the binding site of benzothiadiazine inhibitors (palm I site) in the HCV RdRp (Tarantino et al., 2014). Similarly, the B-site is within the thumb domain and is near to the C-terminal of the hNV RdRp, that is reported to take part in the initiation of RNA replication (Ng et al., 2004). Co-crystallization studies revealed that B-site inhibitors form key interaction with K166, R392, S410, R413, Q414, R419, and C-terminal residue G510. The binding of PPNDS fixes the C-terminal within the active site of hNV RdRp, and therefore obstruct the substrates’ access. The novel site-B residues are highly conserved in viruses of the Caliciviridae family (Smertina et al., 2019). Collectively, the structural similarities shared by the palm site I in HCV, N-pocket in DENV, and site-B in hNV RdRps suggest the opportunity to identify novel small molecules that target this common allosteric pocket, and thus inhibit all of these three pathogenic viruses, with the possibility of inhibiting other RNA viruses.

Currently, there is no approved drug to prevent DENV and hNV infection. The aim of this study is to identify NNIs targeting the common allosteric pocket in RdRps of different RNA viruses. The identified “N-pocket” in DENV RdRp and B-site in hNV RdRp were used as the druggable target. Firstly, a structure-based virtual screening was carried out to examine 39816 commercially available small molecules. Compounds were ranked based on their binding energies with DENV and hNV RdRp, respectively. Top-ranked hits with strong binding energies for both RdRps were considered. The ability of selected compounds to inhibit DENV and hNV RdRp was assessed using *in vitro* enzymatic assay and in viral infection experiments. Among the tested compounds, Entrectinib (RAI-13) demonstrated potent inhibition of both DENV and hNV RdRp. Bio-layer Interferometry (BLI) binding assay confirmed that RAI-13 bound directly to DENV and hNV RdRp. Finally, to evaluate its broad-spectrum antiviral efficacy, we also tested the antiviral activity of RAI-13 on viruses outside of the Flaviviridae and Caliciviridae families. The strategy used in this study should be applicable for identification of broad-spectrum antiviral agents.

## Materials and Methods

### Structure-Based Virtual Screening

The crystal structure of hNV RdRp in complex with the site B inhibitor PPNDS (DPB ID: 4LQ3) and DENV RdRp in complex with the N-pocket inhibitor JF-31-MG46 (PDB ID: 5F3T) were chosen as targets for screening compounds with potential inhibitory effects on both hNV and DENV. Protein structures were prepared for docking using AutoDockTools (ADT, version: 1.5.6) by removing co-crystalized water molecules, adding polar hydrogens, and merging Gasteiger charges (Huey and Morris, 2008).

The active site of NV RdRp was defined as a box of 22 Å × 22 Å × 22 Å, centered near the co-crystalized ligand PPNDS (*x* = 19.644 Å, *y* = 47.352 Å, *z* = 49.301 Å). Correspondingly, the grid center of DENV RdRp located at JF-31-MG46 binding region and was designated at dimensions (*X*, *Y*, and *Z*): 24.332, 52.261, and 45.568 Å. The grid size was set to 22, 22, and 22 Å on *X*, *Y* and *Z* coordinates, respectively. The grid step size for each docking volume was set to 1 Å.

All compounds from TargetMol database were prepared using Open Babel toolkit version 2.3.2 (O’Boyle et al., 2011). Each molecule was assigned hydrogen for pH 7.4, minimized using MMFF94 force field (Halgren, 1996), and converted to PDBQT format used for docking.

Auto Dock Vina software package was used for docking (Seeliger and de Groot, 2010; Trott and Olson, 2010). Total 39816 compounds from the focused database were docked to the defined binding regions of hNV and DENV RdRp, respectively, and ranked by their calculated binding free energies (ΔG_ADV)_. Top hits from virtual screening against each RdRp were visually inspected. Only compounds showed strong binding affinity for both DENV and hNV RdRps were selected for further investigation.

### Molecular Dynamic Simulation

The predicted complexes for RAI-13 docked to hNV and DENV RdRps were embedded in water surroundings and relaxed applying MD simulations for 10 ns (Alonso et al., 2006). All the simulations were carried out utilizing Amber11 software with amber ff99SB force field (Case et al., 2010; Lindorff-Larsen et al., 2010). The parameters of RAI-13 were created using the general Amber force field (GAFF) through the antechamber module (Wang et al., 2001, 2004). The restrained electrostatic potential charges were fitted from quantum mechanism (QM) calculation at B3LYP/6-31G^∗^ level using Gaussian 09 program (Frisch et al., 2014). The LEAP module was used to add protons and solvate polymerase/RAI-13 complexes in a box of TIP3P water, extending at least 10 Å from the complexes. Counter ions were added to hNV-RdRp/RAI-13 and DENV-RdRp/RAI-13 for charge neutralization, respectively. The simulation procedure was performed as previously described (Li et al., 2018), we briefly described here. Before MD simulations, the polymerase/RAI-13 systems were minimized twice. In the first stage, we minimized the positions of water and added ions, while keeping the polymerase/RAI-13 complexes fixed with a force constant 100 kcal mol^–1^ Å^–2^. In the second stage, the entire systems were minimized without any restraints. After minimization, the systems were heated from 0 to 300 K under constant volume and periodic boundary conditions (NVT). Before moving on to the production MD simulation, each system was then equilibrated with weak restrains (10 kcal mol^–1^ Å^–2^) for 500 ps at a constant pressure of 1 atm and temperature of 300 K(NPT). Finally, a length of 10 ns trajectory was computed at 300 K under constant pressure. The time step was set to 2.0 fs throughout the simulation. Particle Mesh Ewald (PME) method and the SHAKE algorithm were applied to treat the Long-range electrostatic interactions and to constrain all bonds that involved hydrogen atoms (Miyamoto and Kollman, 1992).

Snapshots were saved every 0.1 ns and extracted from the MD trajectory. To identify the represent structures of hNV-RdRp/RAI-13 and DENV-RdRp/RAI-13 complexes, we performed cluster analysis of these frames by using kClust module from the MMTSB Tool Set (Feig et al., 2004). The binding free energies and energy decomposition were calculated with molecular mechanics/generalized born surface area (MM/GBSA) method as previously described (Genheden and Ryde, 2015).

### Cell Lines and Viruses

The RAW 264.7 cell line (TIB-71) and the A549 cell line (CCL-185) were purchased from American Type Culture Collection (ATCC) compony, the Huh7.5.1 cell line was obtained from Dr. Rongtuan Lin (McGill University, Montreal, QC, Canada). Cell lines were maintained in Dulbecco’s modified Eagle’s medium (DMEM) (Gibco) supplemented with 10% fetal bovine serum (FBS) at 37°C with 5% CO2.

DENV2 (strain Tr1751) was kindly provided by Dr. Gong Cheng (Center for Infectious Diseases, Tsinghua University, Beijing, China). Jc-1-Gluc HCVcc was kindly provided by Dr. Leiliang Zhang (Institute of Pathogen Biology, Chinese Academy of Medical Sciences, Beijing, China). The MNV strain (SH1603) used in this work was isolated from the feces of BALB/c mice by Dr. Duan’s group, whose genome shows high homology (94%) to that of MNV4 (DQ223043). HIV-1 pseudotyped viruses were produced as we previously described (Guo et al., 2006).

### Chemicals

Small molecules selected from virtual screening procedure were purchased from Target Molecule Corp. PPNDS was purchased from Santa Cruz, 2-(4-methoxy-3-phenyl-phenyl)ethanoic acid (JF-31-MG46) was purchased from MolPort, EZ-Link NHS-LC-LC-Biotin, Biocytin, and Dye PicoGreen were purchased from Thermo Scientific. Polycytidylic acid (5′) potassium(poly(C) were purchased from Sigma–Aldrich(P4903). Vigofect transfection reagent was purchased from Vigorous Biotechnology Beijing Co. The bioassay kits include BCA Protein assay kit (Pierce), RNA extraction kit (Tiandz), one-step SYBR PrimeScript RT-PCR kit (Takara), Cell Counting kit-8 (CCK-8, Beyotime, China). The purity is more than 95% for all compounds that were evaluated in the following experiments.

### Bio-Layer Interferometry (BLI) Binding Assay

The binding affinity of RAI-13 to hNV/DENV RdRps was evaluated by BLI technique using an Octet RED (ForteBio, Inc., CA, United States) instrument. After expression and purification as mentioned below, the hNV/DENV RdRps were mixed with the biotinylation reagent EZ-Link NHS-LC-LC-Biotin and incubated for 1 h. The biotinylated RdRps at 50 μg/ml in kinetics buffer (1 × PBS, 0.002% Tween-20, pH = 7.4) were then captured onto the super streptavidin (SSA) biosensors. Following the immobilization procedure, the biosensors were blocked with biocytin at 5 μg/mL for 1 min and washed with assay buffer. For KD determinations, biosensor tips were immersed into the wells containing 2-fold serial dilution of RAI-13. Each cycle for analysis includes baselines acquisition (60 s), associations of RAI-13 for the determination of K_on_ (60 s), and dissociation of RAI-13 in buffer for the measurement of K_off_ (60 s). The baseline shift was corrected by applying blank binding cycles using buffer only. Parallel experiments were performed for reference sensors that were incubated in assay buffer without polymerase. Finally, a double reference subtraction approach was processed to subtract the influence of the baseline drift and non-specific binding. The binding affinity constant KD (KD = K_off_/K_on_) values were calculated using 1:1 binding model through global fitting of multiple kinetic traces. Data Analysis 9.0 software was used to analyze the real-time monitoring data.

### Expression and Purification of hNV-RdRp and DENV-RdRp

The RdRp gens of NV (Ng et al., 2004) (Norovirus GII.4 Sydney 2012, GenBank: KT239579.2) and RdRp gens of DENV (Yap et al., 2007) (Dengue virus type 3 from Singapore, GenBank: AY662691.1) were synthesized by Suzhou GENEWIZ Biotechnology Co., Ltd., and inserted into pET-21a(+) vector. 1 L LB cultures of Escherichia coli BL21 transfected with pET-21a(+)-NV-RdRp and pET-21a(+)-DENV- RdRp were induced with 0.5 mM IPTG and then respectively grown at 16°C for 18 h and 22°C for 18 h. The cells were collected by centrifugation and resuspended in 10 mL of PBS. Next the harvested cells were lysed by sonication in the presence of lysozyme and DNase I. The lysate was separated by centrifugation at 12000 rpm for 10 min at 4°C. The target proteins were purified by Ni-nitrilotriacetic acid affinity chromatography. Unbound proteins were washed with buffer A (10 mM PBS(PH7.5), 300 mM NaCl, 10%(v/v) glycerol, 2 mM DTT, 10 mM imidazole). The RdRp was eluted by buffer B (10 mM PBS(PH7.5), 300 mM NaCl, 10%(v/v) glycerol, 2 mM DTT, 500 mM imidazole). The RdRp proteins were further purified by size exclusion chromatography. Fractions containing RdRp were dialyzed against buffer C (10 mM PBS(PH7.5), 150 mM NaCl, 2 mM DTT) and concentrated to 3–5 mg/mL. Protein concentration was measured by the BCA Protein assay kit (Pierce). SDS-PAGE analysis was used to indicate the purity of RdRp.

### hNV-RdRp Inhibition Assay

Polymerase activity was measured by detecting the formation of double-stranded RNA (dsRNA) from poly(C), using the fluorescent dye PicoGreen (Singer et al., 1997). RdRp assays were performed in a black 96-well plates, which protects reaction mixture from light. *In vitro* RNA synthesis assays were performed using a single-stranded RNA poly(C) as template and GTP as substrate in a 25 μL reaction mixture. A 0.5 μL of hNV-RdRp (90 μM), dissolved in a reaction buffer containing 10 mM PBS (pH7.5), 2.5 mM MnCl_2_, 5 mM DTT, was incubated with 1 μL of 1 mM compound (PPNDS served as positive control) or the compound vehicle DMSO at 30°C for 10 min. Subsequently, 1 μL of 1 mg/mL poly(C) and 1 μL of 625 μM GTP were added into the mixture for 30 min at 30°C. The final concentrations of the reaction components are 1.8 μM NV-RdRp, 40 ng/mL poly(C), 25 μM GTP, 40 μM compound, 4% DMSO, 10 mM PBS (pH 7.5), 2.5 mM MnCl2 and 5 mM DTT. The reaction was terminated with 10 mM EDTA. After that, 175 μL of fluorescent dye PicoGreen, diluted 200-fold with TE buffer, was added to each well for 5 min at room temperature. The PicoGreen staining and dsRNA quantitation were performed using a microplate reader at standard wavelengths (excitation 480 nm, emission 520 nm) (Urakova et al., 2016).

### DENV-RdRp Inhibition Assay

Polymerase activity was measured by detecting the formation of double-stranded RNA (dsRNA) using the fluorescent dye PicoGreen (Singer et al., 1997). RdRp assays were performed in a black 96-well plates, which protects reaction mixture from light. *In vitro* RNA synthesis assays were performed using a single-stranded RNA poly(C) as template and GTP as substrate in a 25 μL reaction mixture. 5 μL of DENV-RdRp (20 μM), dissolved in a reaction buffer containing 20 mM Tris–HCl (pH 7.4), 2.5 mM MnCl2, 5 mM DTT, was incubated with 1 μL of 1 mM compound (RAI-5, RAI-13, RAI-14, and JF-31-MG46 served as positive control) or the compound vehicle DMSO at 30°C for 10 min. Subsequently, 1 μL of 1 mg/mL poly(C) and 2 μL of 625 μM GTP were added into the mixture for 60 min at 30°C. The final concentrations of the reaction components are 4 μM DENV-RdRp, 40 ng/mL poly(C), 50 μM GTP, 40 μM compound, 4% DMSO, 40 mM Tris–HCl (pH 7.4), 2.5 mM MnCl2 and 5 mM DTT. The reaction was terminated with 10 mM EDTA. After that, 175 μL of fluorescent dye PicoGreen, diluted 200-fold with TE buffer, was added to each well for 5 min at room temperature. Microplate reader was used to quantify the amount of dsRNA at standard wavelengths (excitation 480 nm, emission 520 nm).

### Infection Assays With MNV

2 × 10^5^ per well RAW264.7 cells were seeded into 12-well plates for 24 h, and infected with MNV(SH1603) at a multiplicity of infection (MOI) of 10 in the presence of RAI-13 with 2-fold dilutions (from 0.08 to 20 μM) for 4 h. The culture supernatants were replaced by Fresh medium with RAI-13. Cells were harvested and total RNA was extracted, by using an RNA extraction kit at 48 h.p.i. The RNA extraction kit was used to extract total RNA. The level of viral RNA was determined by qRT-PCR analysis through one-step SYBR PrimeScript RT-PCR kit (Yi et al., 2019). The primer pair (Forward: 5′-CCACTGCTCAGATCACATGC-3′ and Reverse: 5′-TTAGAAAGAAGGCGGCC AGA-3′) amplifies capsid protein of MNV. The Forward and Reverse primers were also, respectively, used for minus-strand and plus-strand RNA reverse transcription. The mouse glyceraldehyde-3-phosphate dehydrogenase (mGAPDH) was served as an internal control and primer pair was designed as following: Forward: 5′-TGCAGTGGCAAAGTGGAGATT-3′ and Reverse: 5′-GTGAGTGGAGTCATACTGGAACA TGT-3′.

### Infection Assays With DENV2

5 × 10^5^ per well A549 cells were seeded into 6-well plates for 24 h, and infected with DENV2 (Tr1751) at an MOI of 0.1 in the presence of RAI-13 with 2-fold dilutions (from 0.08 to 20 μM) for 4 h. The culture supernatants were replaced by Fresh medium with RAI-13 with 2-fold dilutions. Cells were harvested at 48 h.p.i. The RNA extraction kit was used to extract total RNA. The level of viral RNA was determined by qRT-PCR analysis through one-step SYBR PrimeScript RT-PCR kit (Yi et al., 2019). The primer pair (forward: 5′-TCATACTCTATGTGCACAGGAAAG-3′ and reverse: 5′-CGATGAAGCTTGGCCGATAGAACTTCC-3′) amplifies partial envelop protein E gene of DENV2. The Forward and Reverse primers were also, respectively, used for minus-strand and plus-strand RNA reverse transcription. Human glyceraldehyde-3-phosphate dehydrogenase (hGAPDH) was served as an internal control and amplified with primers 5′-ATCATCCCTGCCTC TACTGG-3′ and 5′-GTCAGGTCCACCACTGACAC-3′.

### Cytotoxicity Analysis

Twenty-four hour prior to the cytotoxicity assay, 4 × 10^4^ RAW264.7 or A549 cells were seeded in 96-well plates. After that, 2-fold serial dilutions (from 0.625 to 40 μM) of RAI-13 were added to the cells. Cells treated with DMSO were served as the control. Cells were cultivated for 48 h, after that the cell viability was assessed by Cell Counting kit-8.

### Infection Assay With Jc-1-Gluc HCVcc

2 × 10^4^ per well Huh7.5.1 cells were seeded into 96-well plates for 24 h, and then cells were incubated with RAI-13 (1 μM) and Jc1-Gluc HCVcc at an MOI of 1 for 4 h. Next, the culture supernatants were replaced by Fresh medium with 1 μM RAI-13. After 48 h, we measured the amounts of infectious Jc1 HCVcc in the supernatant by detecting the activity of Gaussia luciferase (Gluc), using a Centro XS3 LB 960 luminometer.

The effect of RAI-13 on Gluc activity was used as negative control. 5 × 10^5^ per well Huh7.5.1 cells were seeded into 6-well plates for 24 h. After that, cells were transfected with pcDNA-Gluc plasmid (500 ng/well) by Vigofect transfection reagent for 4 h. Next, the culture supernatants were replaced by fresh medium with 2-fold serial dilutions (1.25, 2.5, and 5 μM) of RAI-13. After 48 h, we measured the activity of Gaussia luciferase (Gluc) by using a Centro XS3 LB 960 luminometer.

### Infection Assay With HIV-1 Pseudotyped With VSV-GP

The pNL4-3.Luc.R-E- DNA was transfected into HEK293T cell together with plasmid DNA expressing VSV GP. The VSV-G/HIV-Fluc in the culture supernatants was harvested and clarified by centrifugation at 1,000 × *g* for 30 min at 4°C to remove cell debris. The virus samples were aliquoted and stored at −80°C. The selected compound RAI-13 was tested against the HIV-1 pseudotyped viruses in 96-well plates. Briefly, 4 × 10^4^ per well HEK293T cells were seeded into 96-well plates for 24 h. Then HEK293T cells were infected with the VSV-G/HIV-Fluc at an MOI of 0.1 in the presence of RAI-13 at the final concentration of 2 μM. Cells treated with DMSO were served as the control. The infected cells were lysed at 48 h.p.i, firefly luciferase (Fluc) activity was measured by using a Centro XS3 LB 960 luminometer.

## Results

### Validation of the Docking Performance and Accuracy

**FIGURE 1:**
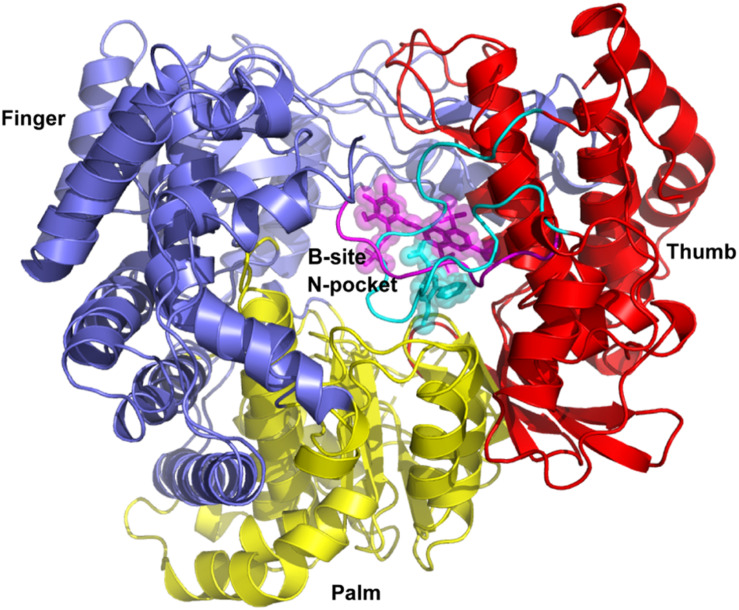
Superimposition of complex structures of DENV-RdRp/JF-31-MG46 (PDB ID 5F3T) and hNV-RpRp/PPNDS (PDB ID 4LQ3). N-pocket inhibitor JF-31-MG46 and B-site ligand PPNDS are shown as cyan and magenta stick representation, respectively. The priming loop in DENV RdRp and C-terminal in hNV RdRp are highlighted in cyan and magenta loops, respectively. DENV and hNV RdRps share an overall structural architecture with fingers (blue), palm (yellow), and thumb domains (red).

The crystal structures for DENV-RdRp/JF-31-MG46 (PDB ID 5F3T) (Noble et al., 2016) and hNV-RpRp/PPNDS (PDB ID 4LQ3) (Tarantino et al., 2014) complexes were chosen as the templates to provide suitable ligand binding pockets for virtual screening. As shown in Figure 1, JF-31-MG46 is located in the N-pocket and is covered by the priming loop in DENV-RdRp. Similarly, PPNDS bound to the B-site in hNV-RpRp and interacted with the C-terminal residues. Superimposition of these two complex structures showed that JF-31-MG46 and PPNDS bound to a common allosteric site. The docking parameters for AutoDock Vina were optimized by redocking the co-crystalized ligands JF-31-MG46 and PPNDS into the inhibitor-binding sites. The calculated binding energies of JF-31-MG46 and PPNDS in their respective binding sites are −7.6 and −8.2 kcal/mol, respectively. Superposition of the redocked and crystallography-determined conformations of JF-31-MG46 and PPNDS leads to RMSD values of only 0.63 and 0.52 Å, respectively (Supplementary Figure S1). Because the binding pockets of PPNDS and JF-31-MG46 are structurally equivalent to each other, we docked PPNDS and JF-31-MG46 into DENV-RdRp and hNV-RdRp, respectively. As seen in Supplementary Figure S2, although the ligands do not match perfectly into the corresponding binding pockets, PPNDS and JF-31-MG46 hold the interaction with the key residues in DENV-RdRp N-pocket and hNV-RdRp B-site, respectively. These results indicate that the docking procedure used in this study is able to describe correct ligand binding pose and suggest a reliable fidelity of docking.

### Structure-Based Virtual Screening

**FIGURE 2:**
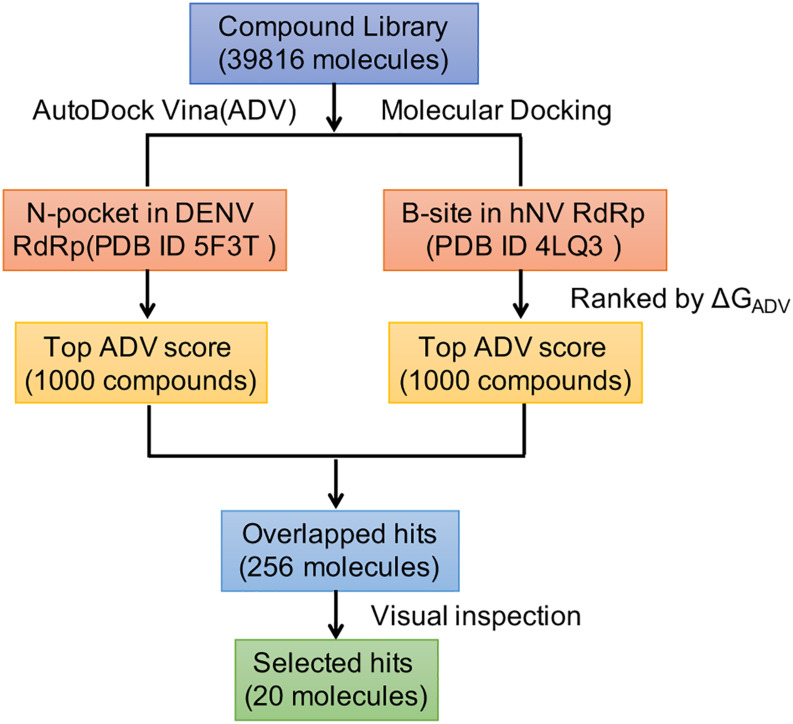
Overall workflow of the structure based virtual screening for identification of inhibitors targeting DENV and hNV RdRps.

**FIGURE 3:**
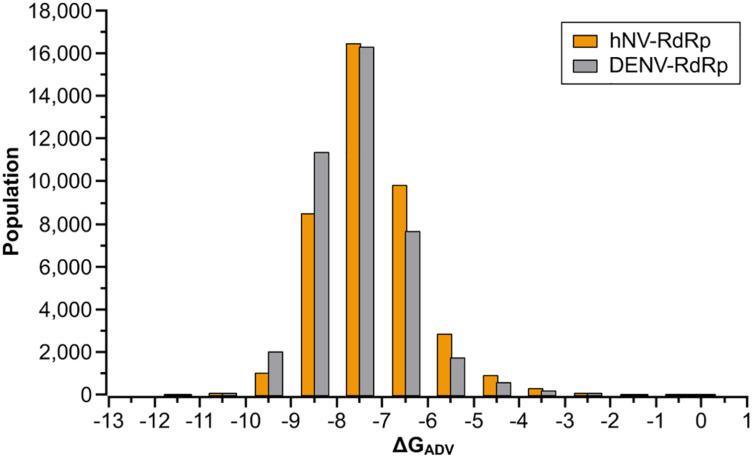
Results of the virtual screening of the TargetMol Database against DENV and hNV RdRps. Bars represent numbers of compounds with predicted free energies of binding in the indicated 1 kcal/mol bins.

To identify compounds that inhibit RdRps of both DENV and hNV, we performed structure-based virtual screening against TargetMol’s Bioactive compound library plus (7568 compounds) and 3D-Biodiversity Library (32248 compounds) by using AutoDock Vina. Compounds in Bioactive compound library have target description and known biological activities, providing great opportunities for drug repurposing. Compounds in 3D-Biodiversity Library have diverse three-dimensional structures, drug-like properties, and are most used to identify new drugs. According to the virtual screening strategy shown in Figure 2, 39816 compounds from TargetMol Database were docked to the identified conserved N-pocket in DENV RdRp and the novel B-site in hNV RdRps. These small molecules were then ranked based on their binding energies (ΔG_ADV_) with the corresponding protein (Figure 3). We then examined the top 1000 compounds for either DENV RdRp or hNV RdRp. There are 256 overlapped compounds. To ensure the quality of compound selection, we then visually inspected these top-ranked hits. Compounds that showed H-bond, π–π, or salt bridge interactions with the corresponding B-site residues among these 256 were retrieved. Finally, 20 compounds were selected for further experimental validation. The calculated binding energies of selected 20 compounds are more favorable than that of PPNDS and JF-31-MG46. Their database ID, CAS number, formula, and molecule weight (MolWt) were listed in Table 1.

**TABLE 1:** The Database ID, molecule name (MolName), CAS, molecule weight (MW), and calculated binding energies [△G_ADV_ (kcal/mol)] of the selected 20 compounds from structure-based virtual screening.

**No.**	**ID**	**MolName**	**CAS**	**MW (g/mol)**	**△G_ADV_ (kcal/mol)**	
					**DENV**	**hNV**
1	T2297	PIK-294	900185-02-6	489.5	–9.7	–9.9
2	T2237	ICG001	847591-62-2	548.6	–9.9	–10.4
3	T3015	Olaparib (AZD2281, Ku-0059436)	763113-22-0	434.5	–9.8	–9.8
4	T0524	Dihydroergotamine Mesylate	6190-39-2	679.8	–9.8	–10.0
5	T3346	AKT inhibitor VIII	612847-09-3	551.6	–9.7	–10.1
6	T4227	SB1317 (TG-02) hydrochloride	937270-47-8	408.9	–9.5	–9.6
7	T6230	Imatinib (STI571)	152459-95-5	493.6	–9.5	–9.5
8	T2374	HTH-01-015	1613724-42-7	468.6	–11.2	–9.7
9	T6453	Conivaptan hydrochloride	168626-94-6	535.0	–9.7	–9.6
10	T6271	Tipifarnib	192185-72-1	489.4	–10.1	–9.4
11	T6110	YM201636	371942-69-7	467.5	–10.2	–10.0
12	T2079	GSK126	1346574-57-9	526.7	–9.5	–10.1
13	T3678	Entrectinib	1108743-60-7	560.6	–9.5	–9.5
14	T6097	GNF-5837	1033769-28-6	535.5	–10.0	–10.2
15	T2640	DCC-2036 (Rebastinib)	1020172-07-9	553.6	–9.9	–10.0
16	T1861	GSK2126458 (Omipalisib)	1086062-66-9	505.5	–9.6	–9.5
17	T1905	EPZ005687	1396772-26-1	539.7	–9.7	–9.5
18	T3057	UNC1999	1431612-23-5	569.7	–9.7	–9.4
19	T6154	SU11274	658084-23-2	568.1	–9.6	–9.5
20	T0486	Irinotecan hydrochloride trihydrate	136572-09-3	677.2	–9.9	–10.6

### Validation of Virtual Screening Hits Using *in vitro* RdRp Inhibition Assay

**FIGURE 4:**
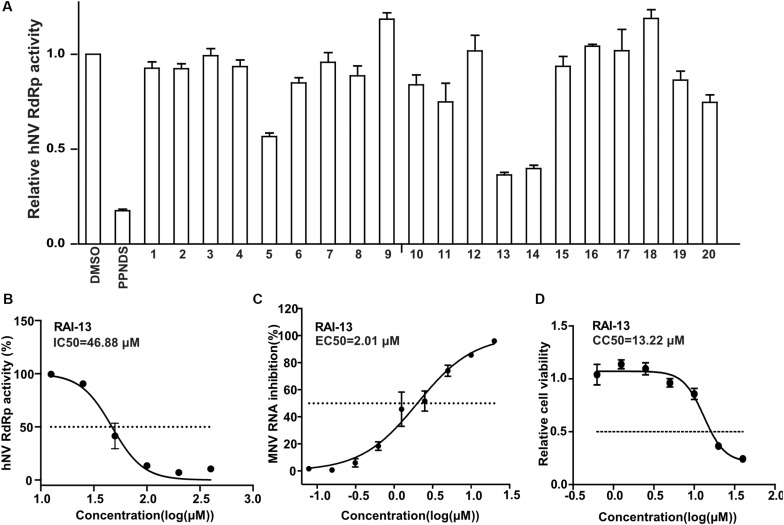
RAI-5, RAI-13 and RAI-14 inhibit the activity of hNV RdRps. **(A)** The inhibitory effect of screened 20 compounds on the activity of hNV RdRps. hNV-RdRp inhibition assay was performed to measure the inhibition of 20 candidate compounds against activity of RdRp *in vitro*. Control groups were treated with DMSO (negative control) and PPNDS, a NV RdRp inhibitor (positive control). An average data of three independent experiments is shown, with the value of control arbitrarily set as 1. **(B)** The inhibitory effect of RAI-13 on hNV RdRp inhibition. Two-fold serially diluted RAI-13 (from 12.5 to 400 μM) were performed in hNV-RdRp inhibition assay. IC50 values were obtained by non-linear regression analysis using GraphPad 5.0. Data are representative of three independent experiments and normalized to control group. **(C)** Anti-MNV activity in cell culture. Two-fold serially diluted RAI-13 (from 0.08 to 20 μM) were incubated with MNV in RAW264.7 cells for 48 h. The inhibition of RAI-13 against MNV replication was measured by detecting MNV RNA through qRT-PCR analysis. EC50 values were obtained by non-linear regression analysis using GraphPad 5.0. The Data are representative of three independent experiments and values are expressed in mean ± SD. **(D)** The cytotoxicity of the RAI-13 was measured using a Cell Counting Kit-8 by following the manufacturer’s instructions, CC50 values were obtained by non-linear regression analysis using GraphPad 5.0. Data shown are means ± s.d. *n* = 3.

We first assessed the inhibitory activity of the selected 20 small compounds against hNV RdRp. PPNDS, a known hNV RdRp inhibitor, was used as the positive control. As shown in Figure 4A, RAI-5, RAI-13,and RAI-14 presented about 50∼70% inhibition of hNV RdRp activity at a concentration of 40 μM. Further examinations showed that RAI-5, RAI-13, and RAI-14 displayed dose dependent inhibition of hNV RdRp, with half maximal inhibitory concentration (IC50) of 47.30 μM for RAI-5, 46.88 μM for RAI-13, and 45.50 μM for RAI-14. The hill slope values are 1.19, 2.94, and 1.88, respectively (Figure 4B and Supplementary Figure S3). To further examine the RAI-13 inhibition of norovirus RNA replication, we infected RAW264.7 cells with MNV(SH1603) in the presence of different concentrations of RAI-5, RAI-13, and RAI-14. Among them, RAI-13 exhibited a dose-dependent inhibition of MNV infection, with an EC50 of 2.01 μM (Figure 4C). In addition to the reduction of total RNA level of MNV, decrease of the plus-strand and minus-strand RNA levels of MNV in infected cells was also detected at a final concentration of 2.5 μM. As seen in Supplementary Figure S4, RAI-13 inhibits the synthesis of both plus-strand and minus-strand RNA of MNV. No cytotoxic effect of RAI-13 was observed as high as 10 μM on RAW264.7 cells (Figure 4D).

**FIGURE 5:**
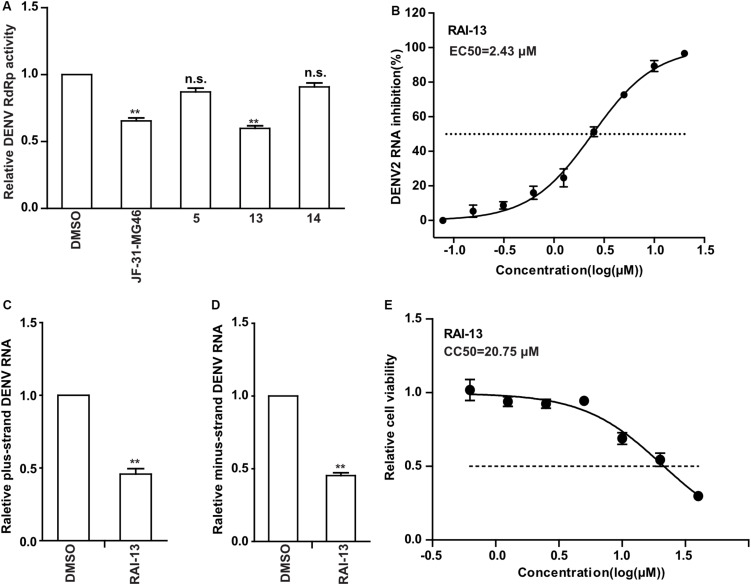
Inhibitory Effects of RAI-13 against DENV RdRp activity and RNA replication. **(A)** The inhibitory effect of RAI-5, RAI-13, and RAI-14 on the activity of DENV RdRps were measured by DENV-RdRp inhibition assay *in vitro* at a concentration of 40 μM. DMSO and JF-31-MG46 were selected as negative and positive control, respectively. The experiments were performed in triplicate. **(B)** Anti-DENV activity in cell culture. Two-fold serially diluted RAI-13 (from 0.08 to 20 μM) were incubated with DENV2 in A549 cells for 48 h. The inhibition of RAI-13 against DENV2 replication was measured by detecting DENV2 RNA through qRT-PCR analysis. EC50 values were obtained by non-linear regression analysis using GraphPad 5.0. The Data are representative of three independent experiments and values are expressed in mean ± SD. **(C,D)** DENV2 infected cells were treated with 2 μM RAI-13. The levels of relative plus-strand and minus-strand DENV2 RNA were measured by qRT-PCR. Error bars indicate standard deviations (*n* = 3). **(E)** The cytotoxicity of the RAI-13 was measured using a Cell Counting Kit-8 by following the manufacturer’s instructions, CC50 values were obtained by non-linear regression analysis using GraphPad 5.0. Data shown are means ± s.d. *n* = 3. *P* values were calculated using a two-sided paired *t*-test. The significance of differences is indicated in the figures (*P < 0.05; **P < 0.01; ***P < 0.001; and n.s., not significant).

To determine whether RAI-5, RAI-13, and RAI-14 could also inhibit DENV RNA replication, we performed the DENV RdRp inhibition experiment using purified DENV RdRp protein. JF-31-MG46 was used as a positive control. The results showed that only RAI-13 markedly inhibited DENV RdRp activity and showed an inhibitory effect equivalent to that of JF-31-MG46, while no inhibitory effect of RAI-5 and RAI-14 were observed on DENV RdRp (Figure 5A). To further examine the RAI-13 inhibition of DENV RNA replication, we infected A549 cells with DENV2 in the presence of different concentrations of RAI-13 and observed a dose-dependent inhibition of DENV2 infection, with an EC50 of 2.43 μM (Figure 5B). In addition to the reduction of total RNA level of DENV2, decrease of the plus-strand and minus-strand RNA levels of DENV2 in infected cells was also detected at a final concentration of 2 μM. These data suggested that RAI-13 inhibits the synthesis of both plus-strand and minus-strand RNA (Figures 5C,D). No cytotoxic effect of RAI-13 was observed as high as 10 μM on A549 cells (Figure 5E). We concluded that RAI-13 decreases DENV RNA synthesis by suppressing the activity of DENV RdRp. Therefore, RAI-13 inhibits the RdRps of both hNV and DENV.

### RAI-13 Binds to hNV and DENV RdRps by BLI

**FIGURE 6:**
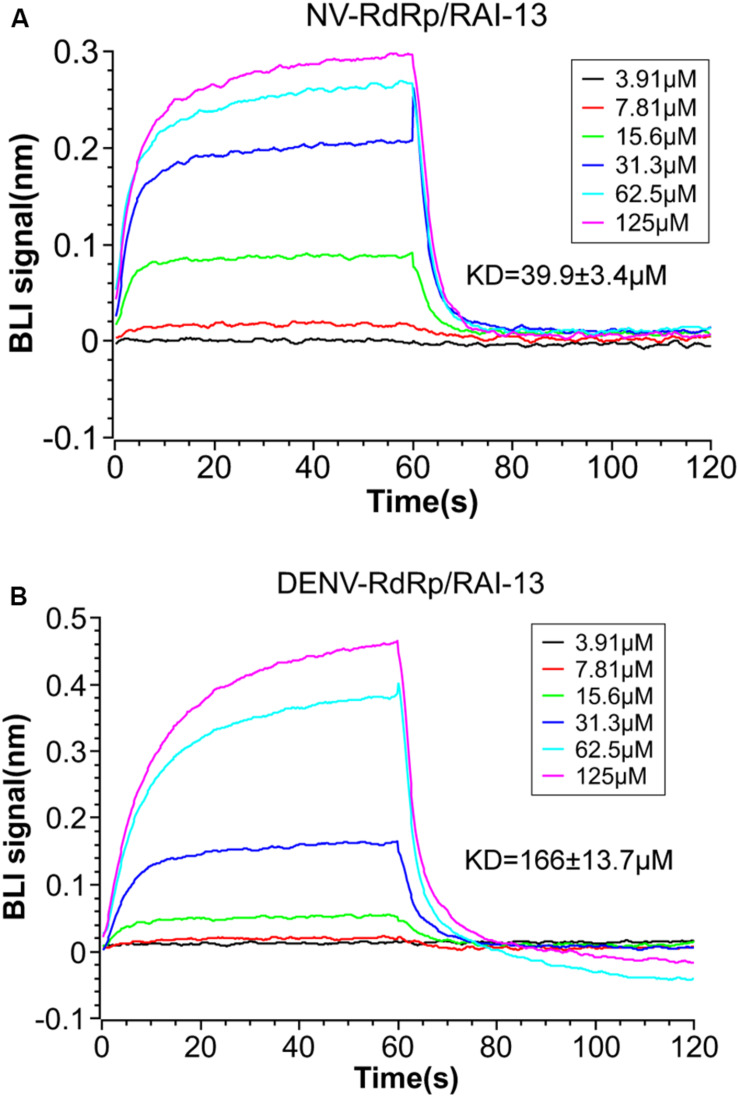
BLI measurement of the interaction between RAI-13 and hNV RdRp **(A)** or DENV RdRp **(B)**. Biotinylated hNV or DENV RdRp was immobilized on SSA biosensors and incubated with 2-fold serially diluted RAI-13 (from 3.91 to 125 μM). Double reference subtraction method was processed to subtract the effect of baseline drift and non-specific binding. KD acquired from fitting into 1:1 binding model by global fitting of multiple kinetic traces and analyzed by Data Analysis 9.0 software. Data shown are representative of three independent experiments.

To determine whether RAI-13 directly binds to the RdRps of hNV and DENV, we performed the BLI binding assay using the ForteBio Octet Red system. The biotinylated hNV or DENV RdRps were captured onto SSA biosensors and applied to solutions containing of 2-fold serially diluted RAI-13 (from 3.91 to 125 μM). The association and dissociation curves were depicted in Figure 6. RAI-13 bound directly to both hNV and DENV RdRps, with fitted KD values of 39.9 and 166 μM, respectively. The fitted KD values were in micromolar range, which were similar to that of JF-31-MG46 (210 μM), a N-pocket inhibitor for DENV RdRp (Noble et al., 2016). Unlike B-site in hNV RdRps that is formed of many charged residues, the N-pocket of DENV RdRps is composed mainly of hydrophobic residues. This may account for the relatively weak binding affinity of DENV RdRps/RAI-13 interaction.

### Binding Poses Analysis of Compound RAI-13

**FIGURE 7:**
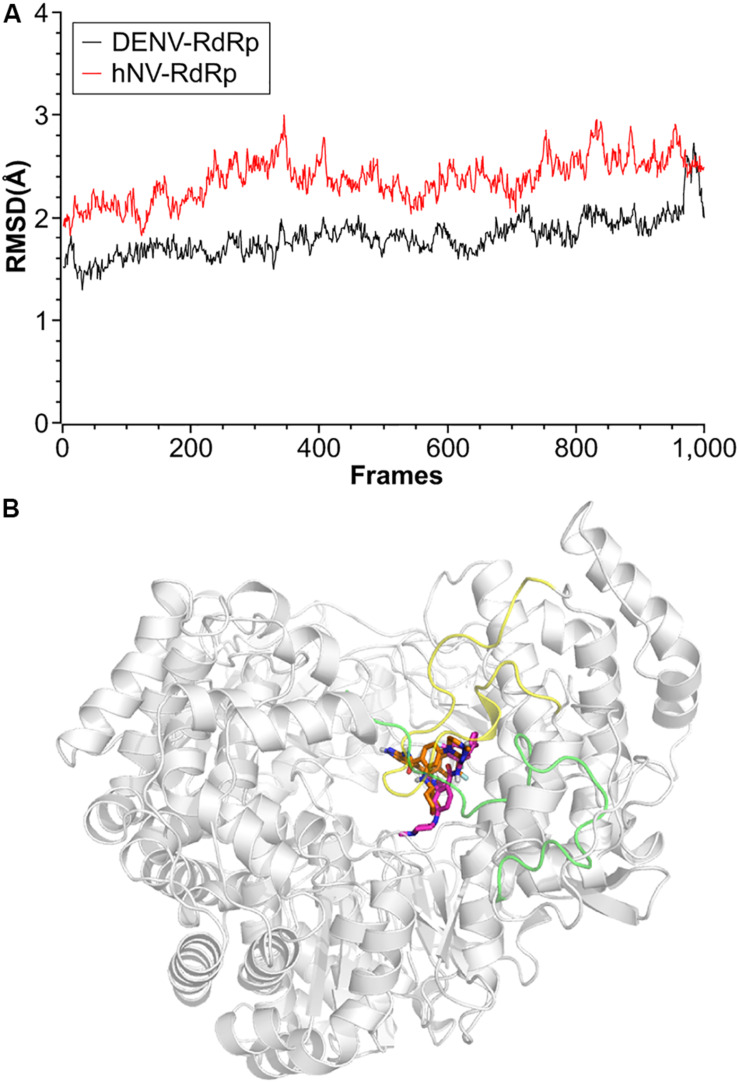
Structural properties of RAI-13 bound to DENV or hNV RdRps during MD simulation over 10 ns. **(A)** Root-mean square deviation (RMSD) values for the backbone atoms. Black and red lines represent complexes of DENV and hNV RdRps, respectively. **(B)** Superimposition of the most representative complex structures of DENV and hNV RdRp bound with RAI-13. The ligand is shown as magentas and orange sticks, respectively. The priming loop in DENV RdRp and C-terminal in hNV RdRp are highlighted in yellow and green loops, respectively.

To better understand the interaction of RAI-13 with DENV or hNV RdRp, we conducted theoretical studies to predict the binding mode. Using the published crystal structures of DENV and hNV RdRps and the AutoDock Vina program, we docked RAI-13 to the ligand binding region of polymerase and obtained the structure of RdRp in complex with RAI-13. The structure was then relaxed using molecule dynamic simulation for 10 ns. After that, MM/GBSA computational method was used to calculate the binding free energy of RAI-13 and decompose it at the amino acid level. As shown in Figure 7, the RMSD indicated great stability of RAI-13 in both DENV RdRp and hNV RdRp binding sites, further supporting RAI-13 as a RdRp inhibitor. Superimposition of the most representative complex structures showed that RAI-13 bound to a common site in the RdRps of hNV and DENV.

**FIGURE 8:**
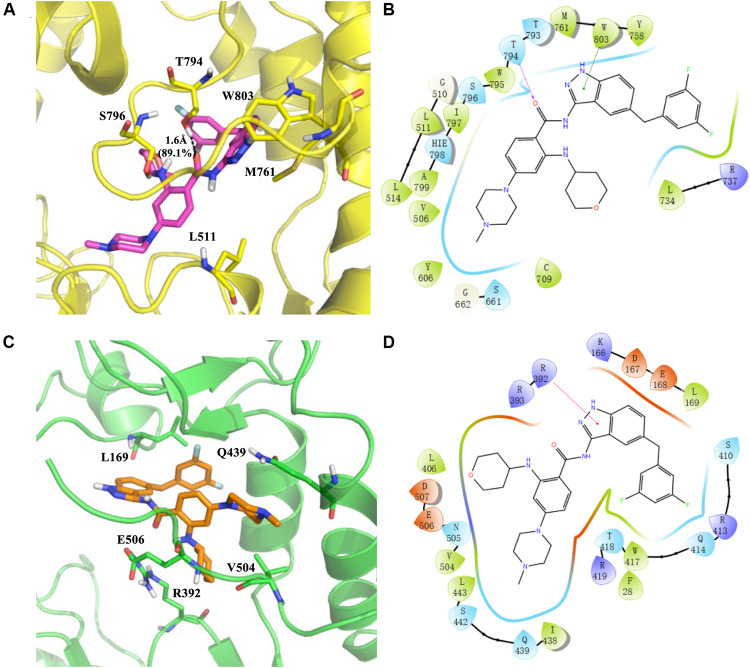
Predicted binding sites for RAI-13 bound to DENV RdRp **(A,B)** and hNV RdRp **(C,D)**. Left panel: The polymerases are shown in cartoon representation with the backbone atoms depicted in yellow (DENV RdRp) and green (hNV RdRp). RAI-13 and the important residues for the polymerase-ligand interactions were shown as sticks. For clarity, only polar hydrogen atoms were depicted in the figures. Right panel: the 2D ligand-polymerase interaction diagrams. Residues within 4 Å of RAI-13 are displayed. Hydrogen bond is represented by the arrows with the distance between the donor and the acceptor. The ⊓⁣−⁣−⁣⊓ stacking and salt bridge are shown in green and purple lines, respectively. Here the color code is that dark blue is positive charged, red is negative charged, light blue is polar, green is hydrophobic, and white is glycine.

RAI-13 is located at the N-pocket of DENV RdRp as expected. As shown by the cavity analysis of the binding site (Supplementary Table S1), the key residues that make main contribution to RAI-13 binding are T794 (−3.25 kcal/mol), W803 (−2.25 kcal/mol), S796 (−1.66 kcal/mol), M761 (−1.57 kcal/mol), L511 (−1.54 kcal/mol), W795 (−1.90 kcal/mol), L514 (−1.57 kcal/mol), A799 (−0.94 kcal/mol), T793 (−0.84 kcal/mol), and I797 (−0.77 kcal/mol). Consistent with the cavity analysis, the main interaction is a very strong hydrogen bond between the carbonyl oxygen atom of the ligand and the hydroxyl group of T794 residue (Figures 8A,B). The percentage occupation of this H-bond is 89.1% during 10 ns MD and the average bond length is 1.6 Å. Moreover, there is also a p - p interaction of the pyrazole ring of RAI-13 with W803. Notable, RAI-13 was anchored by the priming loop (H786-D808) and interacted with the polar residues (T793, S796, and H798) and hydrophobic residues (W795, I797, A799, and W803). The priming loop is one of the special characteristics of flavivirus RdRp and plays a key role in *de novo* RNA initiation. The interaction between RAI-13 and key priming loop residues keeps the polymerase in the closed conformation and, therefore, halts viral RNA synthesis.

As shown in Figures 8C,D, RAI-13 binds to the novel B-site in hNV RdRp that is structurally equivalent to the N-pocket in DENV RdRp. According to the cavity analysis of the binding site (Supplementary Table S2), the main contributing amino acids in the binding cavity are R392 (−3.32 kcal/mol), V504 (−2.05 kcal/mol), Q439 (−2.04 kcal/mol), L169 (−1.98 kcal/mol), E506 (−1.94 kcal/mol), and R419 (−1.90 kcal/mol). The chief interaction is a cationic π-interaction between the indazole ring of RAI-13 with the side chain of R392. Interestingly, RAI-13 interacts with the carboxyl terminus (C-terminus) of NV RdRp, a configuration that is similar to that in DENV RdRp. C-terminal residues such as V504, E506, and D507 make substantial contribution to the binding energy. It has been reported that the C-terminus of NV RdRp also takes part in the initiation of RNA replication. RAI-13 may help to secure the C-terminal end inside the active site and thus hinder the access of both substrates and the ssRNA templates.

In the structure-based virtual screening, DENV-RdRp/JF-31-MG46 and hNV-RdRp/PPNDS complexes were used as the screening templates. It should be noted that RAI-13 has a different molecular skeleton when compared with the reference ligands. As seen in Table 1 and Figure 8, RAI-13 fitted perfectly in both the N-pocket and B-site in DENV and NV RdRps. Correspondingly, the calculated binding energies of RAI-13 in DENV and NV RdRps are more favorable than that of JF-31-MG46 and PPNDS.

### RAI-13 Strongly Inhibits HCV Infection

**FIGURE 9:**
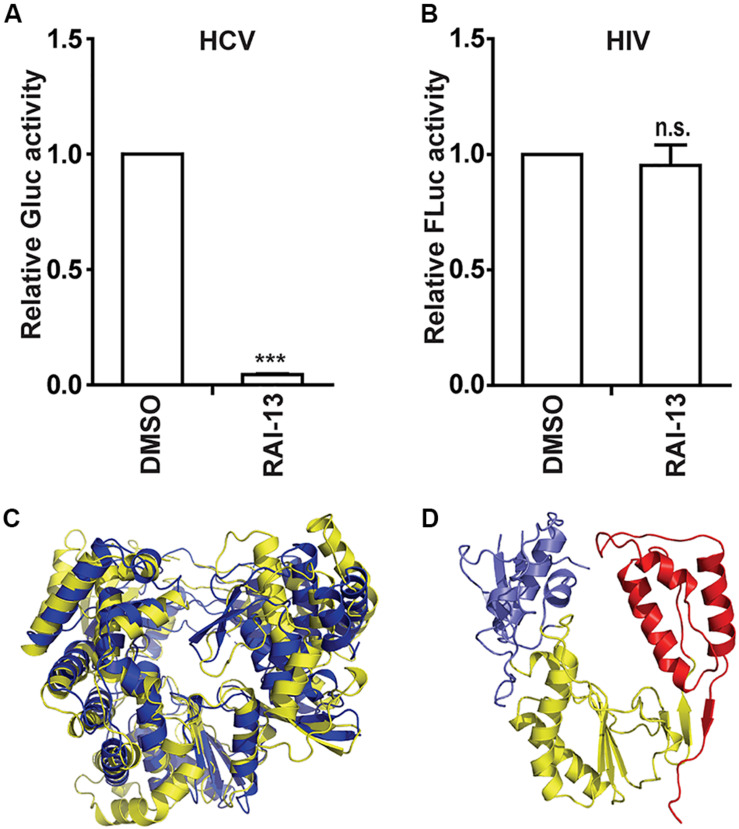
The inhibitory effects of RAI-13 on HCV and HIV-1. **(A)** Jc1-Gluc HCVcc was used to infect Huh7.5.1 cells, which were treated with 1 um RAI-13. Levels of HCV infection were measured by detecting the Gluc activity in cell culture supernatants at 48 hpi. **(B)** VSVG/HIV-Fluc was used to infect HEK293T cells in the presence of 2 μM RAI-13 for 48 h, and then cells were harvested and lysed. Infection of HIV-1 was measured by detecting Fluc activity in the cell lysates. **(C)** Superimposition of the RdRp structures of DENV (yellow) and HCV (blue). **(D)** The polymerase structure of HIV-1 RT-P66 (PDB ID 1HMV). The fingers, palm, and thumb domains were shown in blue, yellow, and red respectively. Data are representative of at least three independent experiments, and values are expressed as means ± SD. Data are normalized to the control group, with the control value arbitrarily set to 1. *P < 0.05; **P < 0.01; ***P < 0.001; and n.s., not significant.

Since both the N-pocket in DENV RdRp and site B in hNV RdRp are structurally equivalent to the palm site I in HCV RdRp, we therefore tested whether RAI-13 is able to inhibit HCV infection. Strikingly, RAI-13 at 1 μM already inhibited Jc1 HCVcc infection by 95% (Figure 9A). We have also assessed the effect of RAI-13 on the Gaussia luciferase (Gluc) activity in Huh7.5.1 cells transfected with a vector expressing Gluc and found no inhibitory effect on Gluc activity at concentrations up to 5 μM of RAI-13 (Supplementary Figure S5). We then tested the inhibition of human immunodeficiency viruses type 1 (HIV-1) and found that even at the highest concentration tested, RAI-13 did not show any inhibition effect (Figure 9B).

To understand the varied sensitivities of different viruses to RAI-13 inhibition, we compared the structures of different viral RNA polymerases. As shown in Figures 9C,D, HCV RdRp shows the closest genetic relatedness and the highest structural similarity to DENV RdRp. They all belong to the Flaviviridae family. In contrast, the HIV-1 reverse transcriptase (HIV-1 RT) has a quite different polymerase folding and is genetically distant from the other RdRps, suggesting that HIV-1 RT is not the right target of RAI-13.

## Discussion

Structure-based virtual screening is a cost-effective and powerful approach, which has been widely used in the early phase of drug discovery (Mastrangelo et al., 2012). After obtaining the 3D structure of a target protein, compounds from certain database are docked into its binding site and screened for biological properties based on calculated binding energies. Utilizing this strategy, we have identified a broad-spectrum inhibitor (RAI-13) of DENV and hNV RdRps. This compound showed better binding energies in the molecular docking procedure and exhibited great stability at its binding region during 10 ns MD simulation. The binding of RAI-13 to the targeted polymerases was further confirmed through *in vitro* binding and cell-based assay. Although this study provides proof-of-concept that inhibiting RdRp activity by targeting the novel allosteric site with small molecule compound, it should be noted that RAI-13 binds to DENV and hNV polymerases with low affinity, and weakly inhibits the polymerase activity of both viruses *in vitro*. The present data warrant further structure-based optimization of RAI-13, which may lead to the development of more potent polymerase inhibitors.

RNA-dependent RNA polymerase is an attractive target for broad-spectrum antiviral therapy because of its essential role in the replication of all RNA viruses (Ahlquist, 2002). As previously shown, the conserved N-pocket in DENV RdRp and the B-site in hNV RdRp are structurally equivalent to each other (Tarantino et al., 2014; Lim et al., 2016; Noble et al., 2016). Compounds that bind to this the common allosteric site may have inhibitory effect against viruses from these two distinct families, Flaviviridae and Caliciviridae, which have many important human pathogens. Figure 9 depicted the detailed ligand-protein interactions of DENV-RdRp/RAI-13 and hNV-RdRp/RAI-13. Visually, both sites are situated within the thumb domain in the analogous position. An interesting observation was that RAI-13 was anchored by the priming loop in DENV RdRps and C-terminal in hNV RdRps, respectively. Both of these two segments are reported to take part in the initiation of RNA replication. In the DENV-RdRp/RAI-13 complex, the priming loop residues (T793, T794, W795, S796, I797, and W804) made the main contribution to the total binding energy. The tight interaction between RAI-13 and the priming loop keep DENV RdRp in a “closed” state conformation and may prevent the polymer from shifting from initiation to elongation stage of replication. In a similar manner, RAI-13 also interacted with the highly conserved residues V504, E506, and D507 on the C-terminus of NV RdRp. The binding of RAI-13 fixes the C-terminal within the active site of hNV RdRp, and therefore obstructs the substrates’ access. Our study indicates that despite the sequence differences between DENV and hNV RdRps, their common allosteric sites share similar geometric property. RAI-13 may be effective against other virus polymerases bearing similar allosteric sites.

Drug repositioning is a promising strategy to develop antiviral agents as new therapeutics (Ashburn and Thor, 2004). Since the safety, tolerability, pharmacokinetics and target of the existing drugs have been thoroughly studied, the time frame of drug development will be greatly reduced. In order to identify DENV and hNV RdRp inhibitors through drug repurposing, we screened compounds from TargetMol Approved Drug Library. Entrectinib, an FDA approved drug for the treatment of ROS1-positive metastatic non-small cell lung cancer and NTRK gene fusion positive solid tumors (Ardini et al., 2016; Russo et al., 2016), has been identified in this study as an inhibitor of DENV, hNV, and HCV. Interestingly, data from our study demonstrate for the first time that Entrectinib may have broad-spectrum antiviral potential. It inhibited hNV and DENV replication activity by targeting viral RdRps. Moreover, we showed that HCV, a member of the Flavivirus family, is extremely sensitive to Entrectinib. The LD50 (Lethal Concentration 50%) of Entrectinib was above 2000 mg/kg (oral, rat). In our study, we did not observe apparent cytotoxicity on RAW264.7 cells or A549 cells after treatments with RAI-13 at concentrations up to 10 μM (Figures 4D, 5E). While further studies need be conducted to characterize the antiviral effect in animal studies. The observation that RAI-13 exhibited more potent antiviral effect in cell than the inhibition of RdRp activity *in vitro*, raise a possibility that RAI-13 may exert an off-target activity against viral infection at cell level, e.g., an indirect effect derived from its inhibition on tyrosine kinases, since RAI-13 (Entrectinib) is a known inhibitor of the tyrosine kinases TRKA/B/C, ROS1, and ALK. Alternatively, cellular environment may be in favor of binding of the inhibitor to RdRp under physiological conditions. In any event, the detailed mechanisms warrant further study.

Our assay showed that RAI-13 has relatively broad antiviral activity, and its antiviral effect is specific to RNA viruses that encode RdRps. For example, RNA viruses such as DENV, HCV, and hNV are inhibited by RAI-13, while the retroviruses HIV-1 was resistant to RAI-13. The difference in sensitivity may due to the polymerase structure divergence between these viruses. As shown in Figure 9, due to the high structural similarities shared by DENV and HCV, RAI-13 inhibited the replication of both viruses. In contrast, the sequence and structure of HIV-1 RT is in dramatic contrast to that of the other three viral polymerases. It is thus not surprising that RAI-13 did not inhibit HIV replication due to the lack of corresponding binding pocket in HIV-1 polymerase. In summary, we have discovered RAI-13 as a potential antiviral drug that targets viral RdRp and may have a broad antiviral effect against many pathogenic RNA viruses.

## Data Availability Statement

The original contributions presented in the study are included in the article/Supplementary Material, further inquiries can be directed to the corresponding author.

## Author Contributions

SC conceptualized the study. DY and QL did the formal analysis and carried out the investigation. LP helped with the resources. ZD and SC supervised the study. YW and YZ contributed to validation. QL wrote the original draft. CL and SC reviewed and edited the manuscript.

## Conflict of Interest

The authors declare that the research was conducted in the absence of any commercial or financial relationships that could be construed as a potential conflict of interest.
