# Supplementary material for: Identification of a Broad-Spectrum Viral Inhibitor Targeting a Novel Allosteric Site in the RNA-Dependent RNA Polymerases of Dengue Virus and Norovirus
Source: Front Microbiol. 2020 Jun 25;11:1440. doi: 10.3389/fmicb.2020.01440 (PMC7330483; doi:10.3389/fmicb.2020.01440)
Supplement: Supplementary file 1 [file Data_Sheet_1.docx]

Supplementary Material


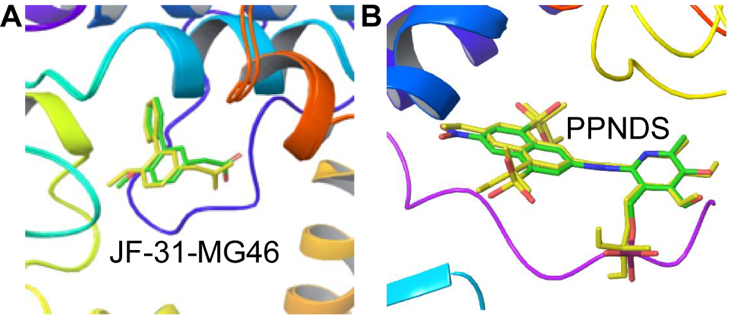


**Supplementary Figure 1.** Superimposition of the redocked (green) and crystallography-determined (yellow) conformations of JF-31-MG46 (**A**) and PPNDS(**B**). Compounds and polymerases were represented in stick and ribbon, respectively.


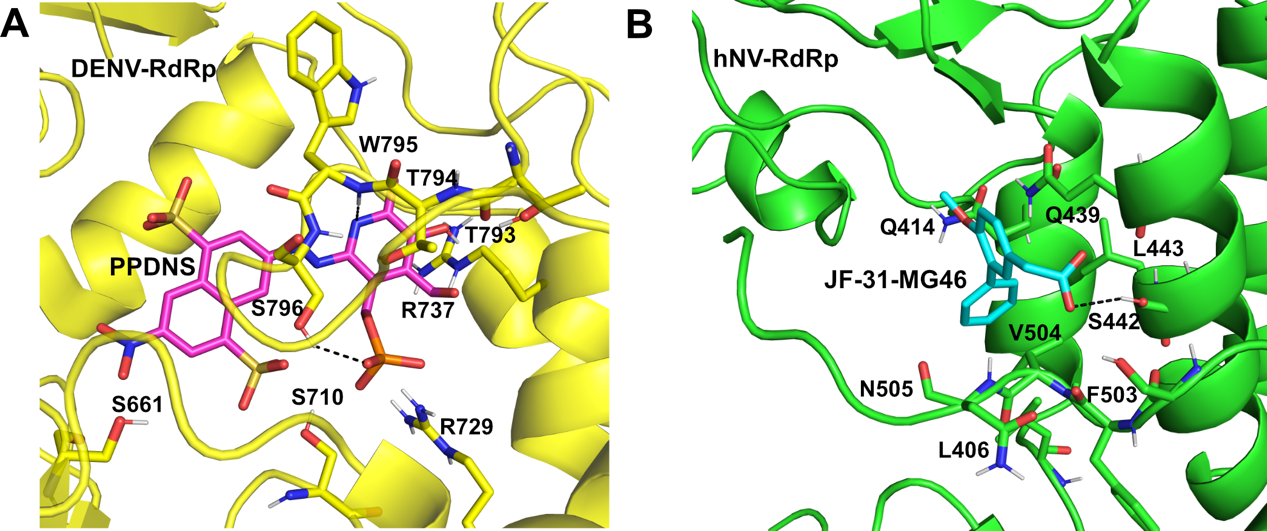


**Supplementary Figure 2.** Predicted binding sites for PPNDS bound to DENV RdRp (A) and JF-31-MG46 bound to hNV RdRp (B). The polymerases are shown in cartoon representation with the backbone atoms depicted in yellow (DENV RdRp) and green (hNV RdRp). PPNDS and JF-31-MG46 are shown shown as sticks with magenta and cyan carbons, respectively.


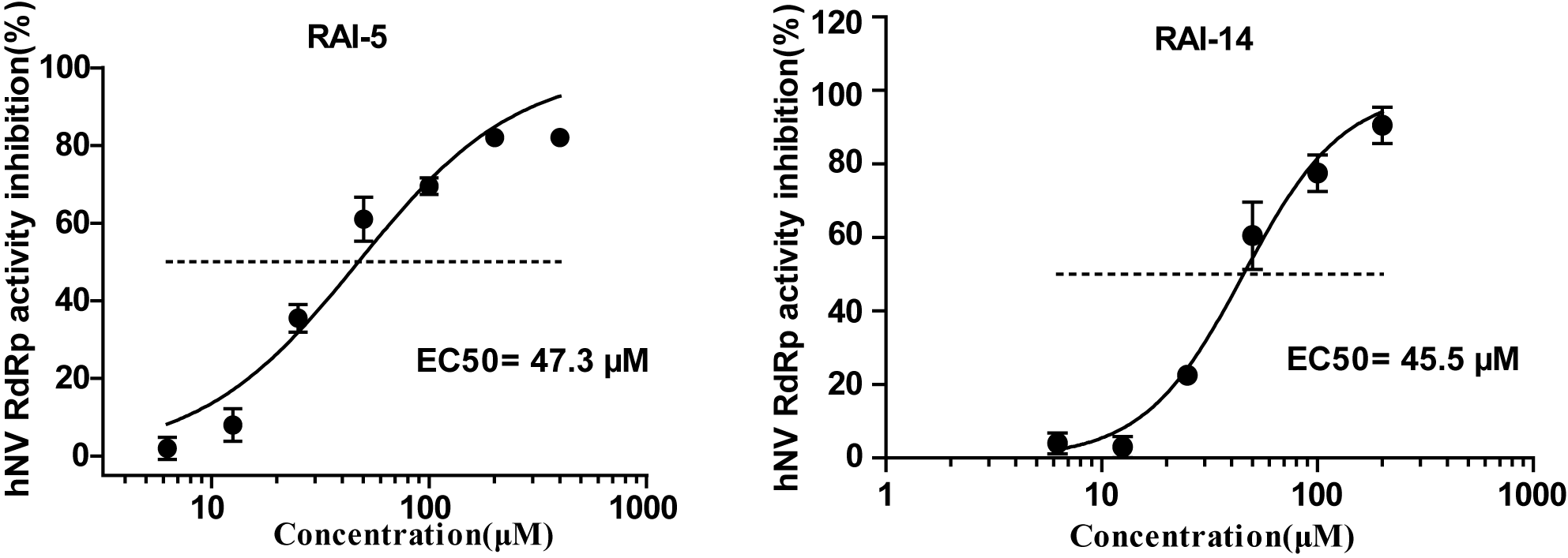


**Supplementary Figure 3.** The inhibitory effect of RAI-5 and RAI-14 on hNV RdRp activity. Two-fold serially diluted RAI-13 (from 6.25 μM to 400 μM) were performed in hNV-RdRp inhibition assay. EC50 values were obtained by nonlinear regression analysis using GraphPad 5.0. Data are representative of three independent experiments and normalized to control group.


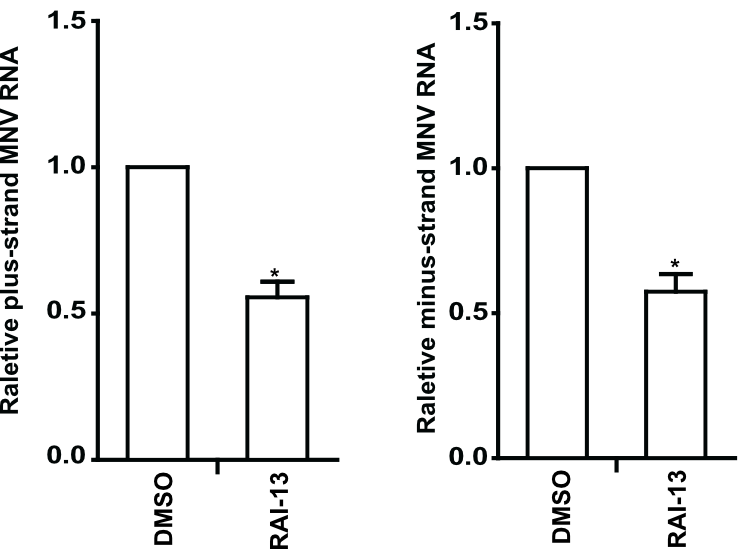


**Supplementary Figure 4.** The plus-strand and minus-strand RNA levels of MNV in infected cells at a final concentration of 2.5 μM of RAI-13. Error bars indicate standard deviations (n = 3). P values were calculated using a two-sided paired t-test. The significance of differences is indicated in the figures (*, P< 0.05; **, P<0.01; ***, P<0.001; and n.s., not significant).


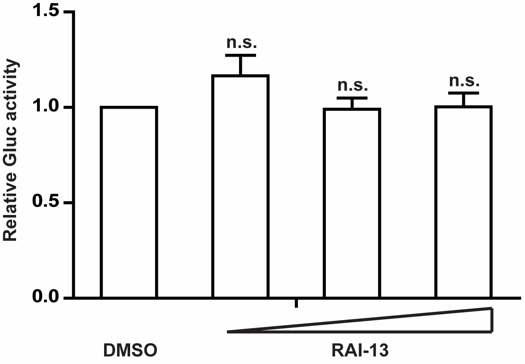


**Supplementary Figure 5.** Effect of RAI-13 on the Gaussia luciferase (Gluc) activity. Huh7.5.1 cells were transfected with pcDNA4.0-Gluc plasmid and incubated with RAI-13 at the final concentrations of 1.25μM, 2.5μM, 5μM. The activity of Gluc in the medium was detected at 48h.p.t. The Data are representative of three independent experiments and values are expressed in mean ± SD.

**Supplementary Table 1. Cavity analysis for the predicted complex of RAI-13 bound to DENV RdRp.** The total energy (kcal mol^-1^) is the sum of the components: van der Waals, Electrostatic, Polar Solvation, and Non-Polar Solvation. The residues are ordered by total energy. Only bind energy better than 0.5 kcal/mol residues are shown.

| Residue | van der Waals | Electrostatic | Polar Solvation | Non-Polar Solv. | TOTAL |
| --- | --- | --- | --- | --- | --- |
| THR 794 | -2.66 | -3.96 | 3.71 | -0.34 | -3.25 |
| TRP 803 | -0.97 | -1.31 | 0.09 | -0.05 | -2.25 |
| SER 796 | -2.16 | -1.12 | 1.89 | -0.27 | -1.66 |
| MET 761 | -1.48 | 0.06 | 0.06 | -0.21 | -1.57 |
| LEU 511 | -1.48 | 0.15 | 0.01 | -0.22 | -1.54 |
| TRP 795 | -1.79 | -0.51 | 1.42 | -0.31 | -1.19 |
| LEU 514 | -0.86 | 0.08 | -0.24 | -0.11 | -1.13 |
| ALA 799 | -0.93 | -0.27 | 0.35 | -0.09 | -0.94 |
| THR 793 | -1.25 | -0.78 | 1.26 | -0.07 | -0.84 |
| ILE 797 | -0.49 | 0.04 | -0.28 | -0.04 | -0.77 |
| ARG 737 | -1.65 | -1.51 | 2.80 | -0.28 | -0.64 |
| CYS 709 | -0.99 | 0.04 | 0.55 | -0.14 | -0.54 |
| SER 661 | -1.14 | -0.32 | 1.09 | -0.13 | -0.51 |
| LEU 734 | -0.46 | -0.01 | 0.06 | -0.10 | -0.51 |

**Supplementary Table 2. Cavity analysis for the predicted complex of RAI-13 bound to hNV RdRp.** The total energy (kcal mol^-1^) is the sum of the components: van der Waals, Electrostatic, Polar Solvation, and Non-Polar Solvation. The residues are ordered by total energy. Only bind energy better than 0.5 kcal/mol residues are shown.

| Residue | van der Waals | Electrostatic | Polar Solvation | Non-Polar Solv. | TOTAL |
| --- | --- | --- | --- | --- | --- |
| ARG 392 | -2.77 | -5.98 | 5.83 | -0.41 | -3.32 |
| VAL 504 | -2.01 | 0.36 | -0.20 | -0.20 | -2.05 |
| GLN 439 | -2.18 | -0.49 | 0.91 | -0.29 | -2.04 |
| LEU 169 | -2.24 | -0.65 | 1.14 | -0.23 | -1.98 |
| GLU 506 | -3.10 | 2.11 | -0.69 | -0.26 | -1.94 |
| ARG 419 | -2.25 | -0.13 | 0.75 | -0.26 | -1.90 |
| LEU 406 | -0.89 | -0.01 | 0.03 | -0.08 | -0.95 |
| GLN 414 | -2.00 | -1.22 | 2.79 | -0.49 | -0.93 |
| THR 418 | -1.09 | -0.03 | 0.27 | -0.05 | -0.91 |
| TRP 417 | -0.98 | -0.58 | 0.73 | -0.03 | -0.86 |
| PHE 28 | -0.96 | -0.52 | 0.86 | -0.23 | -0.85 |
| ARG 393 | -0.94 | -0.50 | 0.87 | -0.07 | -0.65 |
| ARG 413 | -0.42 | -0.93 | 0.85 | -0.13 | -0.64 |
| GLU 168 | -0.91 | -1.37 | 1.78 | -0.11 | -0.61 |
| ASP 507 | -1.33 | -0.51 | 1.38 | -0.15 | -0.61 |
| LEU 443 | -0.65 | 0.06 | 0.01 | -0.03 | -0.61 |
| ASN 505 | -2.00 | 0.09 | 1.56 | -0.24 | -0.58 |
